# One-Flask Synthesis of Pyrazolo[3,4-*d*]pyrimidines from 5-Aminopyrazoles and Mechanistic Study

**DOI:** 10.3390/molecules22050820

**Published:** 2017-05-16

**Authors:** Wan-Ping Yen, Shuo-En Tsai, Naoto Uramaru, Hiroyuki Takayama, Fung Fuh Wong

**Affiliations:** 1 School of Pharmacy, China Medical University, No. 91 Hsueh-Shih Rd., Taichung 40402, Taiwan; a5850478@yahoo.com.tw (W.-P.Y.); u100003044@cmu.edu.tw (S.-E.T.); 2 Program for Biotech Pharmaceutical Industry, China Medical University, No. 91 Hsueh-Shih Rd., Taichung 40402, Taiwan; 3 Department of Environmental Science, Nihon Pharmaceutical University, Komuro Inamachi Kita-adachi-gun, Saitama-ken 10281, Japan; uramaru@nichiyaku.ac.jp; 4 Department of Medico Pharmaceutical Science, Nihon Pharmaceutical University, Komuro Inamachi Kita-adachi-gun, Saitama-ken 10281, Japan; h.takayama@nichiyaku.ac.jp

**Keywords:** pyrazolo[3,4-*d*]pyrimidines, pyrimidines, vilsmeier reaction, heterocyclization, hexamethyldisilazane

## Abstract

A novel one-flask synthetic method was developed in which 5-aminopyrazoles were reacted with *N,N*-substituted amides in the presence of PBr_3_. Hexamethyldisilazane was then added to perform heterocyclization to produce the corresponding pyrazolo[3,4-*d*]pyrimidines in suitable yields. These one-flask reactions thus involved Vilsmeier amidination, imination reactions, and the sequential intermolecular heterocyclization. To study the reaction mechanism, a series of 4-formyl-1,3-diphenyl-1*H*-pyrazol-5-yl-*N,N*-disubstituted formamidines, which were conceived as the chemical equivalent of 4-(iminomethyl)-1,3-diphenyl-1*H*-pyrazol-5-yl-formamidine, were prepared and successfully converted into pyrazolo[3,4-*d*]pyrimidines. The experiments demonstrated that the reaction intermediates were the chemical equivalents of 4-(iminomethyl)-1,3-diphenyl-1*H*-pyrazol-5-yl)formamidines. The rate of the reaction could be described as being proportional to the reactivity of amine reactants during intermolecular heterocyclization, especially when hexamethyldisilazane was used.

## 1. Introduction

**Scheme 1:**
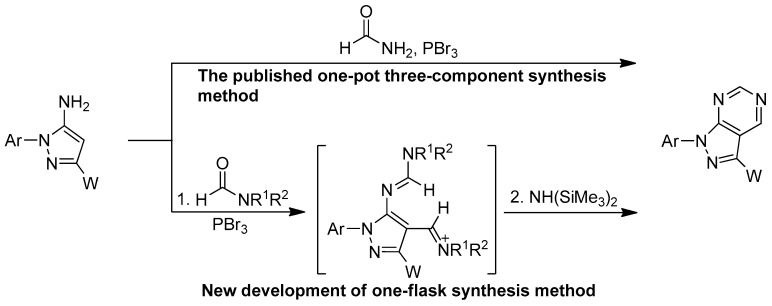
Synthesis of pyrazolo[3,4-*d*]pyrimidines by the different synthetic strategy via the Vilsmeier reaction and intramolecular or intermolecular heterocyclization.

One-flask reactions possess significant advantages and have emerged as a powerful tool in synthetic organic chemistry and reaction design approaches [1,2,3]. The main advantages of using one-flask reactions in organic syntheses are their green chemistry nature and high atom economy due to the lack of workup or the isolation of intermediates involved [4,5,6,7,8,9,10,11,12]. We previously reported an efficient one-pot three-component synthesis of pyrazolo[3,4-*d*]pyrimidines that involved treatment of 5-aminopyrazoles with formamide using PBr3 as the coupling agent (Scheme 1) [13,14,15].

Pyrazolopyrimidine derivatives are important structural moieties, found in pharmacologically active compounds such as a novel series of glucokinase activators [16], antibacterial [17], antifungal [18,19], antioxidant [20], antitumor [21,22,23,24], herbicidal [25], antivirus [26,27], anticancer compounds [28,29], and effective inhibitors of inflammatory mediators in intact cells [30,31]. The pyrazolo[3,4-*d*]pyrimidine core is also isomeric with the biologically significant purine system [32,33]. Numerous synthetic methods were developed for preparing pyrazolopyrimidine derivatives [34,35,36,37]. However, most of these methods are not straightforward and their purification steps are troublesome. Therefore, new and convenient routes for the synthesis of pyrazolo[3,4-*d*]pyrimidine systems have attracted considerable attention [13,14,15].

In this study, we extended our previous one-pot three-component approach for the synthesis of a series of pyrazolo[3,4-*d*]pyrimidine derivatives to develop a novel one-flask synthesis involving Vilsmeier amidination, imination reactions, and sequential intermolecular heterocyclization. First, 5-aminopyrazoles were treated with various Vilsmeier agents, which were generated from the corresponding amide solvents, including *N,N*-dimethylformamide (DMF), *N,N*-diethylformamide (DEF), *N,N*-diisopropylformamide, *N,N*-di-*n*-butylformamide, piperidine-1-carbaldehyde, and pyrrolidine-1-carbaldehyde in the presence of tribromophosphine PBr_3_, to produce the corresponding 4-(iminomethyl)-1,3-diphenyl-1*H*-pyrazol-5-yl-*N,N*-disubstituted formamidine intermediates (Scheme 1) [38,39]. Without isolating the intermediates, we sequentially evaluated the intermolecular heterocyclization reactivity between 4-(iminomethyl)-1,3-diphenyl-1*H*-pyrazol-5-yl-formamidines and amines such as hexamethyldisilazane, hexamethylenetetramine, lithium bis(trimethylsilyl)amine, and sodium bis(trimethylsilyl)amine. These experimental results revealed that commercially available *N,N*-dimethylformamide (DMF)/PBr_3_ and hexamethyldisilazane were the optimal Vilsmeier agent and the promotor, respectively. Specifically, we successfully combined the Vilsmeier amidination and imination reactions with intermolecular heterocyclization to design a high-efficiency one-flask synthesis for the preparation of a series of pyrazolo[3,4-*d*]pyrimidines.

## 2. Results and Discussion

**Scheme 2:**
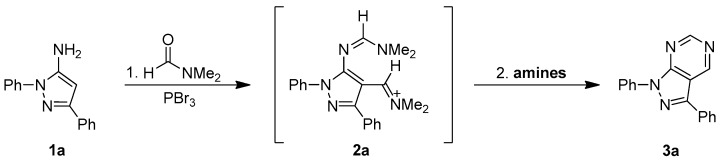
The newly developed one-flask for synthesis of pyrazolo[3,4-*d*]pyrimidine derivatives via Vilsmeier reaction and the sequential heterocyclization.

To optimize the one-flask process for the synthesis of pyrazolo[3,4-*d*]pyrimidine derivatives **3a**–**n** via the sequential Vilsmeier reaction and intermolecular heterocyclization and explain the mechanism the study illustrated in Scheme 2 was performed. 5-Amino-1,3-diphenylpyrazole (**1a**) was prepared by our previously developed method [38,39] and used as the model starting material to improve the intermolecular heterocyclization reaction conditions. Following the reliable published procedure for the Vilsmeier reaction 5-aminopyrazole **1a** was treated with 3.0 equivalent of PBr_3_ in *N,N*-dimethylformamide (DMF) solution at 60 °C for 1.0–2.0 h. The corresponding 4-(iminomethyl)-1,3-diphenyl-1*H*-pyrazol-5-yl-formamidine **2a** was thus obtained in excellent yield (>90%).

Without isolation of intermediate **2a**, various amines, including hexamethyldisilazane (NH(SiMe_3_)_2_), hexamethylenetetramine, lithium bis(trimethylsilyl)amine (LiN(SiMe_3_)_2_), and sodium bis(trimethylsilyl)amine (NaN(SiMe_3_)_2_) were added into the reaction mixture and the solution was heated at reflux for 3–5 h to establish the best heterocyclization conditions (see Table 1). Without the amine agent, only 4-formyl-1,3-diphenyl-1*H*-pyrazol-5-yl-formamidine **7a**, which is the chemical equivalent of 4-(iminomethyl)-1,3-diphenyl-1*H*-pyrazol-5-yl-formamidine intermediate **2a**, was isolated after work-up and purification (see entry 1 in Table 1). Among the amines, the corresponding pyrazolo[3,4-*d*]pyrimidine product **3a** can be produced and isolated in yields ranging from 26% to 91%. Based on the results, we found that commercially available hexamethyldisilazane (NH(SiMe_3_)_2_) provided the best result (91% yield) and the reactivity tendency of the amines was NH(SiMe_3_)_2_ > NaN(SiMe_3_)_2_ > LiN(SiMe_3_)_2_ > hexamethylenetetramine (see the Entries 2–5 in Table 1). We next tried different amounts of NH(SiMe_3_)_2_, including 1.0, 2.0, 3.0, and 4.0 equivalents. The corresponding pyrazolo[3,4-*d*]pyrimidine product **1a** was obtained in 56–91% yield, with the best yield (91%) corresponding to 3 equivalents of (NH(SiMe_3_)_2_) (see the Entries 5 and 6–8 in Table 1). Consequently, we believe that 3.0 equivalent of NH(SiMe_3_)_2_ is the optimum amount for our reaction conditions.

To determine the reactivity of the different Vilsmeier agents (HC(O)NR1R_2_ + PBr_3_), we used different amide solvents, including *N,N*-dimethylformamide (DMF), *N,N*-diethylformamide (DEF), *N,N*-diisopropylformamide, *N,N*-di-*n*-butylformamide, piperidine-1-carbaldehyde, and pyrrolidine-1-carbaldehyde in the presence of 3.0 equivalent of PBr_3_ to prepare the corresponding types of Vilsmeier reagent. Compound **1a** was allowed to react sequentially with these different Vilsmeier reagents at 60 °C for 1.0–2.0 h. When the starting material **1a** was fully consumed, 3.0 equivalents of NH (SiMe_3_)_2_ were added to the reaction mixture which was heated at reflux for 3.0–5.0 h. After the work-up and purification, the corresponding pyrazolo[3,4-*d*]pyrimidine **3a** was obtained in 56–91% yields (see Table 2). Based on the study, commercially available DMF was the best solvent for the preparation of the Vilsmeier reagent in this new one-flask procedure. Based on our optimized experimental results, we believe the most reliable procedure for the one-flask synthesis of pyrazole[3,4-*d*]pyrimidines involves the treatment of 5-aminopyrazole **1a** with 3.0 equivalent of PBr_3_ in DMF solution at 60 °C for 1.0–2.0 h. When the Vilsmeier reaction was completed, the resulting mixture was added with 3.0 equivalents of NH(SiMe_3_)_2_ then heated at reflux at 70 °C to 80 °C for 3.0–5.0 h (monitored by TLC). After work-up and purification by chromatography, the corresponding pyrazolo[3,4-*d*]pyrimidine **3a** was obtained in excellent yield (91%, see Table 2).

Application of the optimized one-flask inter-heterocyclization procedure to 5-amino-1,3-disubstituted pyrazoles **1b**–**i** bearing various N1 substituents, including *o*-Me-Ph, *o*-Cl-Ph, *m*-Me-Ph, *m*-Cl-Ph, *m*-NO_2_-Ph, *p*-Me-Ph, *p*-Cl-Ph, and *p*-Br-Ph, also proceeded smoothly to give the corresponding pyrazolo[3,4-*d*]pyrimidines **3a**–**i** in 78–91% yields (see Table 3).

For further investigation of the effect of the substituent on the C-3 of the pyrazole ring, the same conditions were employed with 5-amino-1-phenyl-3-substituted pyrazoles **1j**–**n** that contained methyl, *t*-butyl, *p*-Me-Ph, *p*-Cl-Ph, or *p*-OMe-Ph groups at the C-3 position of the pyrazole ring. The reaction also proceeded smoothly gave the corresponding products **3j**–**n** in 79–91% yields (see Table 3). All pyrazolo[3,4-*d*]pyrimidines **3a**–**n** were fully characterized by spectroscopic methods and the physical properties and spectroscopic characteristics of the pyrazolo[3,4-*d*]pyrimidines **3a**–**n** were consistent with our published data [13,14,15]. 

For further comparison of the reactivity between this new intermolecular Vilsmeier heterocyclization and the previously published intramolecular heterocyclization method [13,14,15], 5-aminopyrazoles were treated with formamide/PBr_3_. Based on the results of Table 3, the corresponding pyrazolopyrimidines **3a**–**n** were obtained in 78–91% yields by the intermolecular heterocyclization route and in 87–96% yields by intramolecular heterocyclization, respectively. The data suggests that the intramolecular heterocyclization is more favorable as it provided the better isolated yields.

**Scheme 3:**
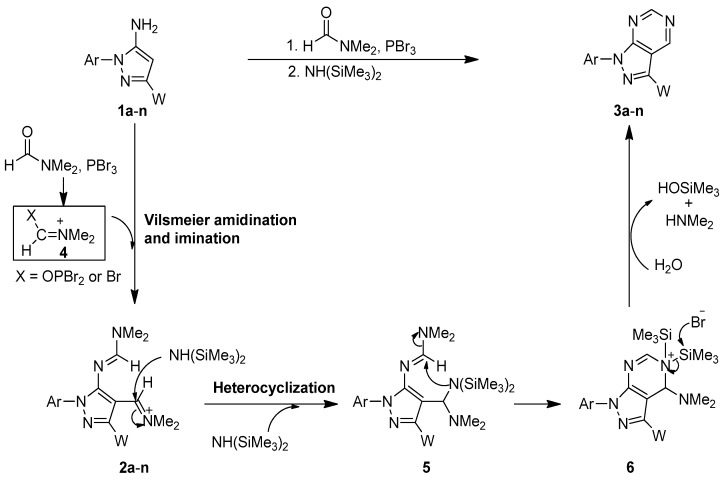
A plausible mechanism for the newly developed one-flask procedure for the synthesis of pyrazolo[3,4-*d*]pyrimidines.

We propose a plausible mechanism for the newly developed one-flask cascade for synthesis of pyrazolo [3,4-*d*]pyrimidines as shown in Scheme 3. Initiallly, *N,N*-dimethylformamide (DMF) reacted with the coupling agent PBr_3_ to form the Vilsmeier reactive species **4** in situ [40,41,42,43,44]. Sequentially, 5-amino-1,3-disubstituted pyrazoles **1a**–**n** reacted with the reactive species **4** to undergo the amidination and imination reaction to give the 1*H*-pyrazol-5-yl-*N,N*-disubstituted formamidine intermediates **2a**–**n** (see Scheme 3). When the Vilsmeier reaction was complete (by monitoring TLC), NH(SiMe_3_)_2_ was directly added into the reaction mixture to perform the substitution reaction with the imino group to generate intermediate **5**. A sequential intermolecular heterocyclization reaction then took place to produce intermediate **6**. After the desilylation reaction occurred caused by bromide anion and water, the corresponding pyrazolo[3,4-*d*]pyrimidines **3a**–**n** were obtained in good yields.

To further study the mechanism, 4-formyl-1,3-diphenyl-1*H*-pyrazol-5-yl-*N,N*-dimethyl formamidine **7a** was synthesized [20] and reacted with various amines including NH(SiMe_3_)_2_, hexamethylenetetramine, LiN(SiMe_3_)_2_, and NaN(SiMe_3_)_2_, to carry out the intermolecular heterocyclization. The heterocyclization was successfully and smoothly underwent to give pyrazole[3,4-*d*]pyrimidine product **3a** in 37–91% yields. Particularly, NH(SiMe_3_)_2_ was most efficient base for heterocyclization to afford the desired product in 91% yield (see Entry 4 in Table 4). The similar reactivity tendency of heterocyclization was observed in this study: NH(SiMe_3_)_2_ > NaN(SiMe_3_)_2_ > LiN(SiMe_3_)_2_ > hexamethylenetetramine (see Entries 1–4 in Table 4). 4-formyl-1,3-diphenyl-1*H*-pyrazol-5-yl-*N,N*-disubtituted formamidines **7b**–**e** with grafting the different amino-substituent on amidinyl groups, such as NEt_2_, *N*(*i*-Pr)_2_, *N*(*n*-Bu)_2_, and piperidinyl, were then allowed to reacted with NH(SiMe_3_)_2_ in DMF solution at reflux to give the corresponding pyrazolo[3,4-*d*]pyrimidine **3a** for the investigation of the reactivity of substrates. Based on the experimental result, among of starting substrates **7b**–**e** displayed the good to excellent reactivity in heterocyclization, except for **7c** possessing the bulky *N* (*i*-Pr)_2_ substituent moiety on amidinyl groups (see Entries 1 and 5–8 in Table 4). Furthermore, 4-formyl-1,3-diphenyl-1*H*-pyrazol-5-yl-*N,N*-dimethyl formamidine **7a** with the NMe_2_ substituent on amidinyl groups was the best suitable reactant in the intermolecular heterocyclization (91%, see Table 4). The above results also gave more proof to our proposed mechanism, for example, the new one-flask reaction would take place through 4-(iminomethyl)-1*H*-pyrazol-5-yl-formamidine intermediates **2a**–**n**. On the other hands, the commercial available *N,N*-dimethylformamide (DMF) in the presence of PBr_3_ and hexamethyldisilazane were the best Vilsmeier agent and the promoted cyclization base.

## 3. Experimental Section

### 3.1. General Information

All chemicals were reagent grade and used as purchased. All reactions were carried out under nitrogen atmosphere and monitored by TLC analysis. Flash column chromatography purification of compounds was carried out by gradient elution using hexanes in ethyl acetate (EA) unless otherwise stated. Commercially available reagents were used without further purification unless otherwise noted. ^1^H-NMR were recorded at 200, 400, or 500 MHz and ^13^C-NMR recorded at 50, 100, or 125 MHz, respectively, in CDCl_3_, CH_3_OD, and DMSO-*d*_6_ as solvent (see supplementary materials). The standard abbreviations s, *d*, *t*, *q*, and *m* refer to singlet, doublet, triplet, quartet, and multiplet, respectively. Coupling constant (*J*), whenever discernible, have been reported in Hz. Infrared spectra (IR) were recorded as neat solutions or solids; and mass spectra were recorded using electron impact or electrospray ionization techniques. The wavenumbers reported are referenced to the polystyrene 1601 cm^–1^ absorption. High-resolution mass spectra were obtained by means of a JMS-HX110 mass spectrometer (JEOL, Tokyo, Japan). 

### 3.2. Standard Procedure for the Synthesis of Pyrazolo[3,4-d]pyrimidines ***3a–n***

The optimized procedure involved the treatment of 5-aminopyrazoles **1a**–**n** (1.0 equiv) with PBr_3_ (~3 equiv.) in various amide solutions including *N,N*-dimethylformamide (DMF), *N,N*-diethylformamide (DEF), *N,N*-diisopropylformamide, *N,N*-di-*n*-butylformamide, piperidine-1-carbaldehyde, or pyrrolidine-1-carbaldehyde (2 mL) at 50–60 °C for 1.0–2.0 h. When the reaction was completed (as monitored by TLC), an amine such as hexamethyldisilazane (NH(SiMe_3_)_2_), hexamethylenetetramine, lithium bis(trimethylsilyl)amine (LiN(SiMe_3_)_2_), or sodium bis(trimethylsilyl)amine (NaN(SiMe_3_)_2_) was added into the reaction mixture which was stirred at reflux for 3–5 h. When the intermolecular heterocyclization was complete, the resulting mixture was added to saturated sodium bicarbonate (15 mL) and extracted with dichloromethane (15 mL × 2). The organic extracts were dried over MgSO_4_, filtered, and concentrated under reduced pressure. The residue was purified by column chromatography on silica gel to give the corresponding pyrazolo[3,4-*d*]pyrimidines **3a**–**n** in 69–91% yields.

*1,3-Diphenyl-1H-pyrazolo[3,4-d]pyrimidine* (**3a**) [13,14,15,45]. Light-yellow solid; m.p. 158–159 °C (hexane–EtOAc). ^1^H-NMR (CDCl_3_, 400 MHz): δ 7.34–7.38 (1H, m, ArH), 7.48–7.51 (1H, m, ArH), 7.53–7.57 (4H, m, ArH), 8.06 (2H, d, *J* = 8.00 Hz, ArH), 8.31 (2H, d, *J* = 8.00 Hz, ArH), 9.12 (1H, s), 9.51 (1H, s). ^13^C-NMR (CDCl_3_, 100 MHz,): δ 114.24, 121.46 (2 × C), 126.86, 127.39 (2 × C), 129.24 (4 × C), 129.64, 131.50, 138.50, 145.00, 152.82, 153.34, 155.61. IR (KBr): 1632, 1586, 1554, 1497, 1366, 1219 cm^–1^. EIMS *m*/*z*: 272 (M^+^, 100), 273 (18), 271 (31), 142 (11), 77 (34), 69 (24), 51 (11).

*1-(2-Methylphenyl)-3-phenyl-1H-pyrazolo[3,4-d]pyrimidine* (**3b**) [13,14,15]. Light-yellow solid; m.p. 140–141 °C (hexane–EtOAc). ^1^H-NMR (CDCl_3_, 400 MHz): δ 2.48 (3H, s, CH_3_), 7.18 (1H, d, *J* = 7.60 Hz, ArH), 7.42–7.45 (1H, m, ArH), 7.50 (1H, d, *J* = 8.00 Hz, ArH), 7.54–7.58 (2H, m, ArH), 8.05–8.10 (2H, m, ArH), 9.12 (1H, s), 9.50 (1H, s). ^13^C-NMR (CDCl_3_, 100 MHz): δ 21.6 (CH_3_), 114.18, 118.77, 122.17, 127.42 (2 × C), 127.77, 129.06, 129.23 (2 × C), 129.61, 131.55, 138.38, 139.33, 144.92, 152.80, 153.32, 155.60. IR (KBr): 1636, 1497, 1223, 1096 cm^–1^. EIMS *m*/*z*: 286 (M^+^, 100), 287 (20), 285 (19), 77 (10).

*1-(2-Chlorophenyl)-3-phenyl-1H-pyrazolo[3,4-d]pyrimidine* (**3c**) [13,14,15]. Yellow solid; m.p. 139–140 °C (hexane–EtOAc). ^1^H-NMR (CDCl_3_, 400 MHz): δ 7.48–7.51 (3H, m, ArH), 7.53–7.57 (2H, m, ArH), 7.60–7.64 (2H, m, ArH), 8.05 (2H, d, *J* = 8.00 Hz, ArH), 9.08 (1H, s), 9.54 (1H, s). ^13^C-NMR (CDCl_3_, 100 MHz): δ 113.01, 125.54, 127.38 (2 × C), 127.72, 128.38, 129.65 (2 × C), 130.08, 130.82, 131.40, 132.19, 134.74, 145.70, 152.87, 154.50, 155.91. IR (KBr): 3012, 1636, 1582, 1497, 1362, 1223, 1084 cm^–1^. EIMS *m*/*z*: 306 (M^+^, 96), 308 (28), 307 (M^+^ + 1, 15), 272 (15), 271 (100), 195 (11), 77 (42), 75 (10), 51 (11).

*1-(3-Methylphenyl)-3-phenyl-1H-pyrazolo[3,4-d]pyrimidine* (**3d**) [13,14,15]. Yellow solid; m.p. 80–81 °C (hexane–EtOAc). ^1^H-NMR (CDCl_3_, 400 MHz): δ 2.52 (3H, s, CH_3_), 7.18 (1H, d, *J* = 8.00 Hz, ArH), 7.41–7.45 (1H, m, ArH), 7.49 (1H, d, *J* = 7.20 Hz, ArH), 7.53–7.57 (2H, m, ArH), 8.05–8.10 (4H, m, ArH), 9.16 (1H, s), 9.53 (1H, s). ^13^C-NMR (CDCl_3_, 100 MHz): δ 21.60, 114.15, 118.70, 122.11, 127.38 (2 × C), 127.72, 129.02, 129.19 (2 × C), 129.57, 131.52, 138.36, 139.28, 144.86, 152.75, 153.27, 155.55. IR (KBr): 1632, 1613, 1585, 1493, 1420, 1366, 1265 cm^–1^. EIMS *m*/*z*: 286 (M^+^, 100), 287 (22), 285 (21), 77 (9).

*1-(3-Chlorophenyl)-3-phenyl-1H-pyrazolo[3,4-d]pyrimidine* (**3e**) [13,14,15]. White solid; m.p. 185–186 °C (hexane–EtOAc). ^1^H-NMR (CDCl_3_, 400 MHz): δ 7.31 (1H, d, *J* = 8.00 Hz, ArH), 7.44–7.50 (2H, m, ArH), 7.52–7.57 (2H, m, ArH), 8.32–8.42 (2H, m, ArH), 9.14 (1H, s), 9.49 (1H, s). ^13^C-NMR (CDCl_3_, 100 MHz): δ 114.51, 118.90, 121.07, 126.61, 127.40 (2 × C), 129.25 (2 × C), 129.85, 130.21, 131.17, 134.95, 139.58, 145.62, 152.88, 153.61, 155.73. IR (KBr): 1585, 1555, 1489, 1404, 1366, 1312, 1215, 1088 cm^–1^. EIMS *m*/*z*: 306 (M^+^, 100), 308 (32), 307 (M^+^ + 1, 26), 305 (22), 77 (16).

*1-(3-Nitrophenyl)-3-phenyl-1H-pyrazolo[3,4-d]pyrimidine* (**3f**) [13,14,15]. Yellow solid; m.p. 179–180 °C (hexane–EtOAc). ^1^H-NMR (CDCl_3_, 400 MHz): δ 7.54–7.61 (3H, m, ArH), 7.73 (1H, dd, *J* = 8.0, 16.4 Hz, ArH), 8.08 (2H, d, *J* = 8.00 Hz, ArH), 8.19 (1H, d, *J* = 8.00 Hz, ArH), 8.87 (1H, d, *J* = 8.00 Hz, ArH), 9.20 (1H, s), 9.37 (1H, s), 9.55 (1H, s). ^13^C-NMR (CDCl_3_, 100 MHz): δ 114.74, 115.70, 120.87, 126.04, 127.51 (2 × C), 129.36 (2 × C), 130.16, 130.18, 130.88, 139.64, 146.08, 148.87, 153.14, 154.00, 156.05. IR (KBr): 1636, 1528, 1489, 1346, 1003 cm^–1^. EIMS *m*/*z*: 317 (M^+^, 100), 318 (17), 77 (14).

*1-(4-Methylphenyl)-3-phenyl-1H-pyrazolo[3,4-d]pyrimidine* (**3g**) [13,14,15]. Brown solid; m.p. 133–134 °C (hexane–EtOAc). ^1^H-NMR (CDCl_3_, 400 MHz): δ 2.39 (3H, s, CH_3_), 7.30 (2H, d, *J* = 8.00 Hz, ArH), 7.45 (1H, d, *J* = 8.00 Hz, ArH), 7.50 (2H, dd, *J* = 7.2, 14.8 Hz, ArH), 8.00 (2H, d, *J* = 8.00 Hz, ArH), 8.11 (2H, d, *J* = 8.00 Hz, ArH), 9.07 (1H, s), 9.43 (1H, s). ^13^C-NMR (CDCl_3_, 100 MHz): δ 20.98, 113.92, 121.21 (2 × C), 127.18 (2 × C), 128.76 (2 × C), 129.37, 129.61 (2 × C), 131.43, 135.94, 136.56, 144.47, 152.58, 152.95, 155.33. IR (KBr): 1636, 1589, 1516, 1386, 1219, 1088 cm^–1^. EIMS *m*/*z*: 286 (M^+^, 100), 287 (22), 285 (28), 77 (10). HRMS Calcd. for C_18_H_14_N_4_: 286.1218; Found: 286.1216.

*1-(4-Chlorophenyl)-3-phenyl-1H-pyrazolo[3,4-d]pyrimidine* (**3h**) [13,14,15]. Yellow solid; m.p. 147–148 °C (hexane–EtOAc). ^1^H-NMR (CDCl_3_, 400 MHz): δ 7.48–7.51 (3H, m, ArH), 7.53–7.57 (2H, m, ArH), 8.03 (2H, d, *J* = 8.00 Hz, ArH), 8.32 (2H, d, *J* = 8.00 Hz, ArH), 9.11 (1H, s), 9.49 (1H, s). ^13^C-NMR (CDCl_3_, 100 MHz): δ 114.32, 122.24 (2 × C), 127.37 (2 × C), 129.23 (2 × C), 129.28 (2 × C), 129.78, 131.24, 132.11, 137.13, 145.23, 152.89, 153.36, 155.66. IR (KBr): 1632, 1555, 1497, 1215, 1054 cm^–1^. EIMS *m*/*z*: 306 (M^+^, 100), 308 (31), 307 (M^+^ + 1, 23), 305 (17), 77 (14).

*1-(4-Bromophenyl)-3-phenyl-1H-pyrazolo[3,4-d]pyrimidine* (**3i**) [13,14,15]. Yellow solid; m.p. 180–181 °C (hexane–EtOAc). ^1^H-NMR (CDCl_3_, 400 MHz): δ 7.51–7.59 (3H, m, ArH), 7.66 (2H, d, *J* = 8.40 Hz, ArH), 8.05 (2H, d, *J* = 8.00 Hz, ArH), 8.29 (2H, d, *J* = 8.00 Hz, ArH), 9.13 (1H, s), 9.51 (1H, s). ^13^C-NMR (CDCl_3_, 100 MHz): δ 114.37, 119.99, 122.53 (2 × C), 127.37 (2 × C), 129.23 (2 × C), 129.79, 131.22, 132.24 (2 × C), 137.63, 145.29, 152.88, 153.40, 155.67. IR (KBr): 1586, 1555. 1481, 1400, 1389, 1215, 1072 cm^–1^. EIMS *m*/*z*: 350 (M^+^, 100), 352 (M^+^ + 2, 99), 353 (15), 351 (27), 194 (14), 77 (30).

*3-Methyl-1-phenyl-1H-pyrazolo[3,4-d]pyrimidine* (**3j**) [13,14,15,46]. Brown solid; m.p. 77–78 °C (hexane–EtOAc). ^1^H-NMR (CDCl_3_, 400 MHz): δ 2.70 (3H, s, CH_3_), 7.32 (1H, dd, *J* = 7.20, 14.80 Hz, ArH), 7.50–7.79 (2H, dd, *J* = 7.60, 15.60 Hz, ArH), 8.19 (2H, d, *J* = 8.00 Hz, ArH), 9.07 (1H, s), 9.16 (1H, s). ^13^C-NMR (CDCl_3_, 100 MHz): δ 12.59, 115.79, 121.07 (2 × C), 126.49, 129.22 (2 × C), 138.50, 143.35, 151.77, 152.77, 155.70. IR (KBr): 3240, 1643, 1503, 1439, 1211 cm^–1^. EIMS *m*/*z*: 210 (M^+^, 100), 211 (16), 209 (27), 195 (13), 142 (15), 77 (37), 69 (11), 57 (16), 55 (13), 51 (13).

*3-tert-Butyl-1-phenyl-1H-pyrazolo[3,4-d]pyrimidine* (**3k**) [13,14,15]. Yellow solid; m.p. 45–46 °C (hexane–EtOAc). ^1^H-NMR (CDCl_3_, 400 MHz): δ 1.57 (9H, s, *t*-Bu), 7.28–7.32 (1H, m, ArH), 7.51 (2H, dd, *J* = 7.60, 15.60 Hz, ArH), 8.22 (2H, d, *J* = 8.00 Hz, ArH), 9.04 (1H, s), 9.32 (1H, s). ^13^C-NMR (CDCl_3_, 100 MHz): δ 30.05 (3 × C), 34.51, 114.02, 121.17 (2 × C), 126.33, 129.13 (2 × C), 138.66, 152.84, 153.16, 154.88, 155.04. IR (KBr): 3048, 2967, 2666, 1636, 1578, 1508, 1427, 1366, 1188, 1096 cm^–1^. EIMS *m*/*z*: 252 (M^+^, 43), 238 (18), 237 (100), 222 (12), 105(11), 77(17), 57(11).

*3-(4-Methylphenyl)-1-phenyl-1H-pyrazolo[3,4-d]pyrimidine* (**3l**) [13,14,15]. White solid; m.p. 138–139 °C (hexane–EtOAc). ^1^H-NMR (CDCl_3_, 400 MHz): δ 2.44 (3H, s, CH_3_), 7.36 (3H, d, *J* = 6.80 Hz, ArH), 7.55 (2H, dd, *J* = 8.00, 16.00 Hz, ArH), 7.95 (2H, d, *J* = 8.00 Hz, ArH), 8.30 (2H, d, *J* = 8.00 Hz, ArH), 9.12 (1H, s), 9.49 (1H, s). ^13^C-NMR (CDCl_3_, 100 MHz): δ 21.39, 114.24, 121.41 (2 × C), 126.74, 127.23 (2 × C), 128.64, 129.18 (2 × C), 129.87 (2 × C), 138.51, 139.77, 145.06, 152.79, 153.27, 155.52. IR (KBr): 3117, 1582, 1501, 1223, 1092 cm^–1^. EIMS *m*/*z*: 286 (M^+^, 100), 287 (21), 285 (26).

*3-(4-Chlorophenyl)-1-phenyl-1H-pyrazolo[3,4-d]pyrimidine* (**3m**) [13,14,15,45]. Light-yellow solid; m.p. 194–193 °C (hexane–EtOAc). ^1^H-NMR (CDCl_3_, 400 MHz): δ 7.37 (1H, dd, *J* = 7.60, 15.20 Hz, ArH), 7.51–7.57 (m, 4 H, ArH), 8.00 (2H, d, *J* = 8.00 Hz, ArH), 8.28 (2H, d, *J* = 8.00 Hz, ArH), 9.13 (1H, s), 9.47 (1H, s). ^13^C-NMR (CDCl_3_, 100 MHz): δ 114.03, 121.47 (2 × C), 127.01, 128.51 (2 × C), 129.27 (2 × C), 129.48 (2 × C), 129.98, 135.71, 138.43, 143.85, 152.60, 153.37, 155.69. IR (KBr): 1632, 1555, 1504, 1404, 1219, 1092 cm^–1^. EIMS *m*/*z*: 306 (M^+^, 100), 308 (33), 307 (M^+^ + 1, 26), 305 (22), 77 (10).

*3-(4-Methoxylphenyl)-1-phenyl-1H-pyrazolo[3,4-d]pyrimidine* (**3n**) [13,14,15]. Light-yellow solid; m.p. 169–170 °C (hexane–EtOAc). ^1^H-NMR (CDCl_3_, 400 MHz): δ 3.86 (3H, s, OCH3), 7.04 (2H, d, *J* = Hz, ArH), 7.33 (1H, dd, *J* = 7.60, 14.80 Hz, ArH), 7.52 (2H, dd, *J* = 7.60, 15.60 Hz, ArH), 7.96 (2H, d, *J* = 8.00 Hz, ArH), 8.28 (2H, d, *J* = 8.00 Hz, ArH), 9.08 (1H, s), 9.43 (1H, s). ^13^C-NMR (CDCl_3_, 100 MHz): δ 55.36 (OCH_3_), 114.13, 114.57 (2 × C), 121.26 (2 × C), 124.01, 126.61, 128.61 (2 × C), 129.14 (2 × C), 138.51, 144.74, 152.66, 153.18, 155.43, 160.72. IR (KBr): 3059, 1632, 1613, 1528, 1501, 1431, 1362, 1300, 1258, 1219, 1173, 1092 cm^–1^. EIMS *m*/*z*: 302 (M^+^, 100), 303 (22), 287 (23), 77 (15).

## 4. Conclusions

We have successfully developed the one-flask method to synthesize pyrazolo[3,4-*d*]pyrimidines by treating 5-amino-pyrazoles, in presence of PBr_3_ coupling agent and then hexamethyldisilazane. In this new one-flask reaction was contained Vilsmeier reaction and the sequential intermolecular heterocyclization two steps. Based on the improved studies of the different type of Vilsmeier agents and amines, we found the commercial available DMF/PBr_3_ and hexamethyldisilazane were the best Vilsmeier agent and the efficient base for this newly developed one-flask synthesis. For the mechanistic study, 4-(iminomethyl)-1,3-diphenyl-1*H*-pyrazol-5-yl-*N,N*-disubstituted formamidines were demonstrated as the reaction intermediates by using a series of 4-formyl-1,3-diphenyl-1*H*-pyrazol-5-yl-*N,N*-disubstituted formamidines successfully reacted with amines to give pyrazolo[3,4-*d*]pyrimidines due to they were conceived as the chemical equivalent species. On the other hands, the order of reactivity of amines in intermolecular heterocyclization was NH(SiMe_3_)_2_ > NaN(SiMe_3_)_2_ > LiN(SiMe_3_)_2_ > hexamethylenetetramine. Through the further comparison variation reactive study between intramolecular and intermolecular Vilsmeier heterocyclization reaction, we found the intramolecular heterocyclization be able to provide the better results.

## Figures and Tables

**Table 1:** The study of amine agents in the one-flask for synthesis of pyrazolo[3,4-*d*]pyrimidines.

Entry	Amine Agents	Equiv.	Yields (%) of Compound 3a
1	Without base	-	- ^a^
2	Hexamethylenetetramine	3	33
3	Lithium bis(trimethylsilyl)amine (LiN(SiMe_3_)_2_)	3	67
4	Sodium bis(trimethylsilyl)amine (NaN(SiMe_3_)_2_)	3	81
5	Hexamethyldisilazane (NH(SiMe_3_)_2_)	3	91
6	Hexamethyldisilazane (NH(SiMe_3_)_2_)	1	56
7	Hexamethyldisilazane (NH(SiMe_3_)_2_)	2	63
8	Hexamethyldisilazane (NH(SiMe_3_)_2_)	4	75

^a^ 1*H*-pyrazol-5-yl-*N*,*N*-disubstituted formamidine **2a** was isolated.

**Table 2:**
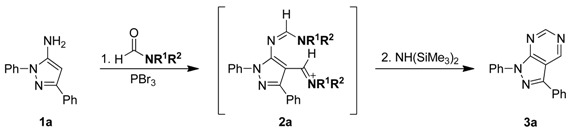
The study of the reactivity of the different Vilsmeier agents in the one-flask synthesis of pyrazole[3,4-*d*]pyrimidines.

Entry	Amide Solvents	Yields (%) of Compound 3a
1	*N*,*N*-dimethylformamide (DMF)	91
2	*N*,*N*-diethylformamide (DEF)	86
3	*N*,*N*-diisopropylformamide	83
4	*N*,*N*-di-*n*-butylformamide	81
5	piperidine-1-carbaldehyde	69
6	pyrrolidine-1-carbaldehyde	56

**Table 3:**
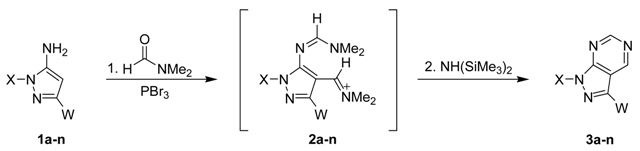
The results of the one-flask synthesis of pyrazolo[3,4-*d*]pyrimidines from 5-aminopyrazoles, DMF/PBr_3_ and NH(SiMe_3_)_2_.

Substrates	X	W	No.	Yields of 3a–n (%)	
				Intermolecular Reaction	Intramolecular Reaction ^a^
**1a**	Ph	Ph	**3a**	91	96
**1b**	*o*-Me-Ph	Ph	**3b**	78	93
**1c**	*o*-Cl-Ph	Ph	**3c**	86	91
**1d**	*m*-Me-Ph	Ph	**3d**	89	92
**1e**	*m*-Cl-Ph	Ph	**3e**	91	92
**1f**	*m*-NO_2_-Ph	Ph	**3f**	87	87
**1g**	*p*-Me-Ph	Ph	**3g**	91	-
**1h**	*p*-Cl-Ph	Ph	**3h**	87	91
**1i**	*p*-Br-Ph	Ph	**3i**	81	95
**1j**	Ph	Me	**3j**	79	93
**1k**	Ph	*t*-Bu	**3k**	87	91
**1l**	Ph	*p*-Me-Ph	**3l**	84	93
**1m**	Ph	*p*-Cl-Ph	**3m**	88	91
**1n**	Ph	*p*-OMe-Ph	**3n**	91	94

^a^ the reported data has been published and cited in ref. [13,14,15].

**Table 4:**
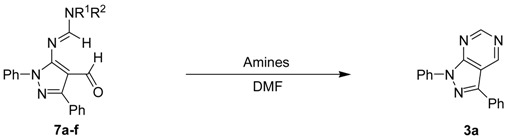
The mechanistic study for the intermolecular heterocyclization from 4-formyl-1,3-disubstituted-1*H*-pyrazol-5-yl-formamidines **7a**–**n** with various amines.

Entry	Substrates	NR^1^R^2^	Amines	Yields of 3a (%)	
1	**7a**	NMe_2_	Hexamethylenetetramine	**3a**	37
2	**7a**	NMe_2_	LiN(SiMe_3_)_2_	**3a**	51
3	**7a**	NMe_2_	NaN(SiMe_3_)_2_	**3a**	84
4	**7a**	NMe_2_	NH(SiMe_3_)_2_	**3a**	91
5	**7b**	NEt_2_	NH(SiMe_3_)_2_	**3a**	89
6	**7c**	N(*i*-Pr)_2_	NH(SiMe_3_)_2_	**3a**	61
7	**7d**	N(*n*-Bu)_2_	NH(SiMe_3_)_2_	**3a**	81
8	**7e**	Piperidinyl	NH(SiMe_3_)_2_	**3a**	86

## Supplementary Materials

Supplementary materials are available online.
