# Supplementary material for: One-Flask Synthesis of Pyrazolo[3,4-d]pyrimidines from 5-Aminopyrazoles and Mechanistic Study
Source: Molecules. 2017 May 16;22(5):820. doi: 10.3390/molecules22050820 (PMC6154590; doi:10.3390/molecules22050820)

Supporting Information

One-Flask Synthesis of Pyrazolo[3,4-*d*]pyrimidines from 5-Aminopyrazoles and Mechanistic Study

Wan-Ping Yen [1,2], Shuo-En Tsai [1,2], Naoto Uramaru [3], Hiroyuki Takayama [4] and Fung Fuh Wong [1,*]

Table of Contents

NMR Spectra of Reported Compounds..S2

3a

1,3-Diphenyl-1*H*-pyrazolo[3,4-*d*]pyrimidine

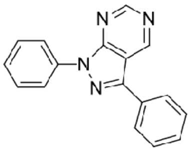

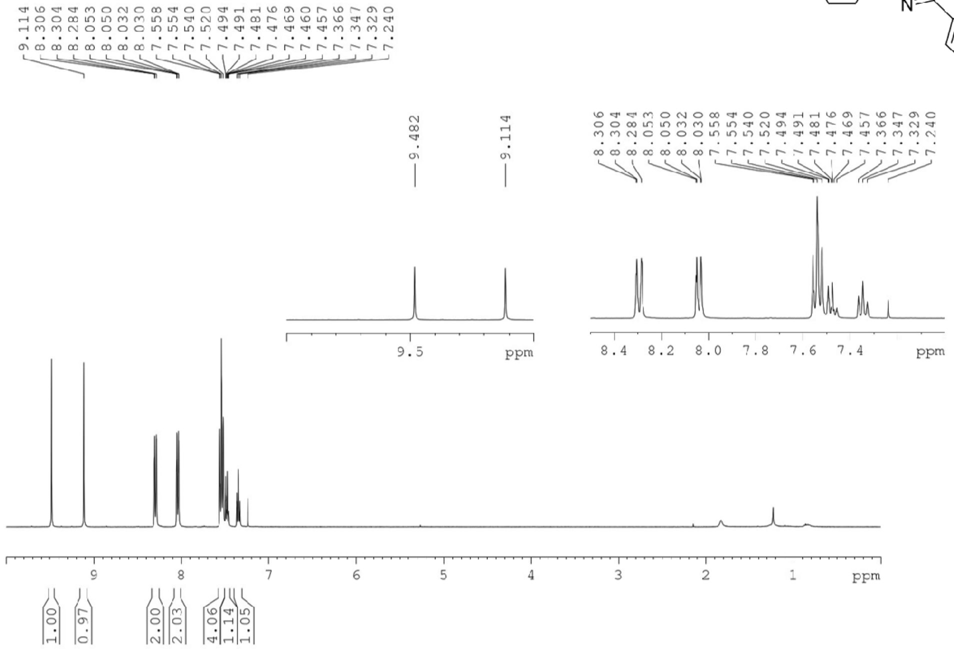

3a

1,3-Diphenyl-1*H*-pyrazolo[3,4-*d*]pyrimidine

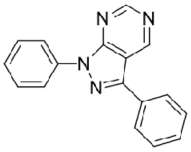

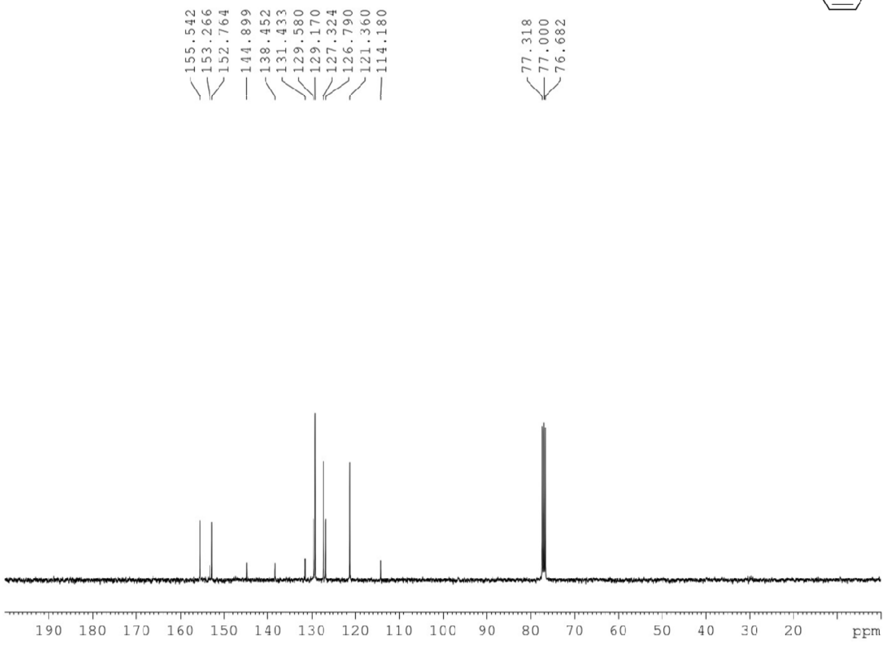

3b

1-(2-Mcthylphcnyl) -3-phcnyl-1*H*-pyrazolo[3,4-*d*]pyrimidinc

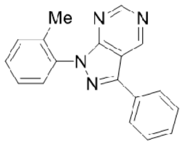

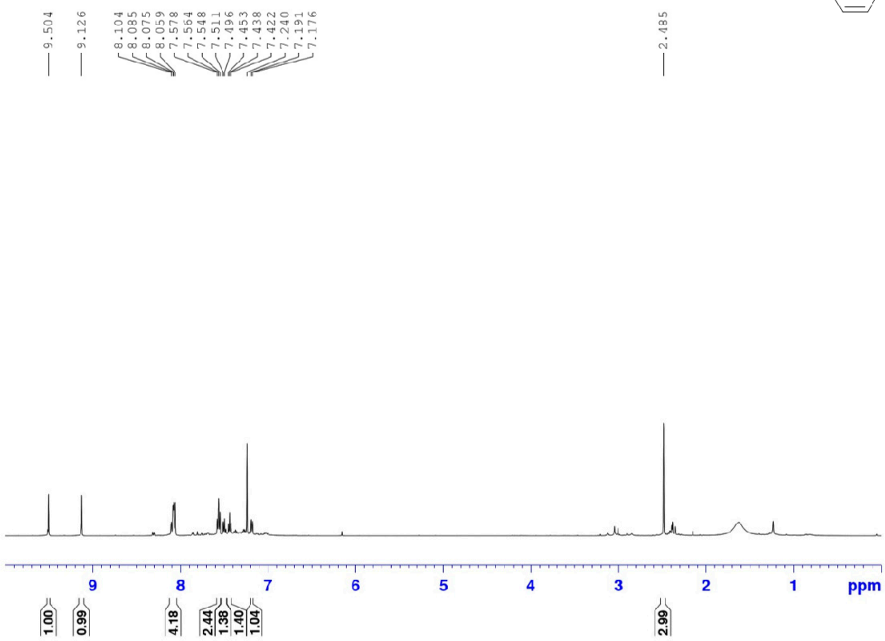

3b

1-(2-Methylphenyl) -3-phenyl-1*H*-pyrazolo[3,4-*d*]pyrimidine

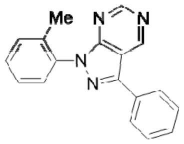

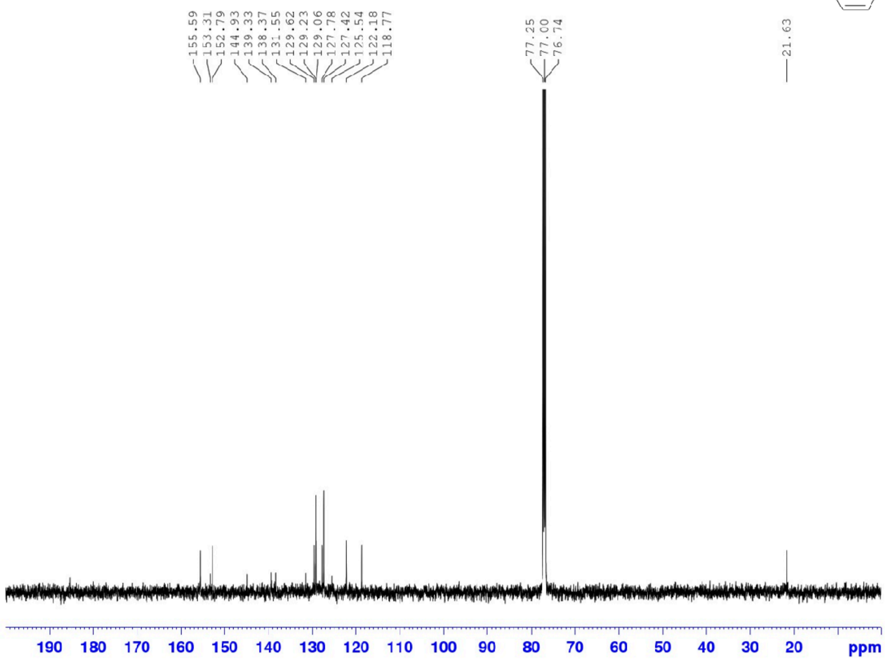

3c

1-(2-chlorophenyl) -3-phenyl-1*H*-pyrazolo[3,4-*d*]pyrimidine

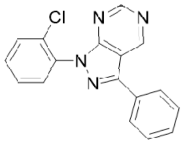

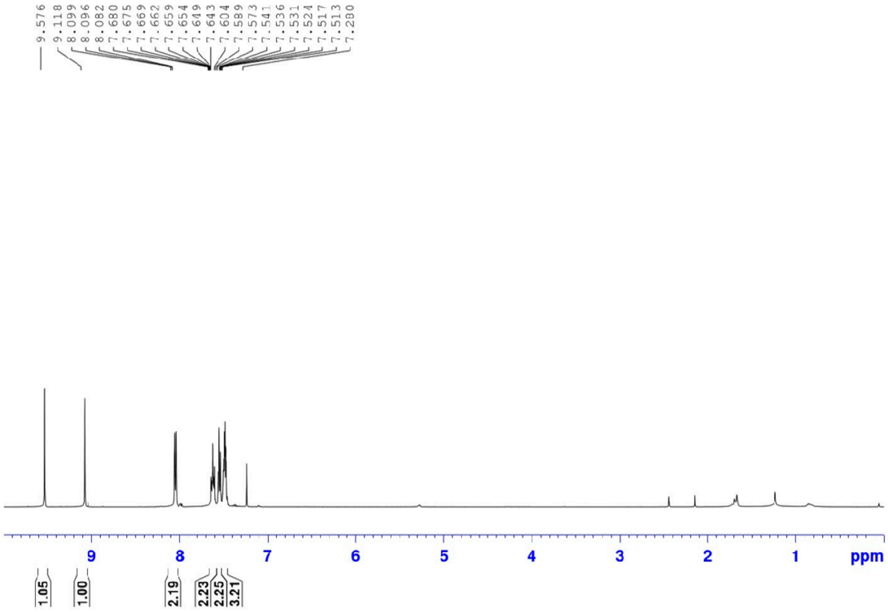

3c

1-(2-chlorophenyl) -3-phenyl-1*H*-pyrazolo[3,4-*d*]pyrimidine

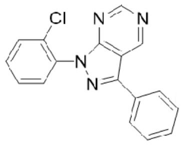

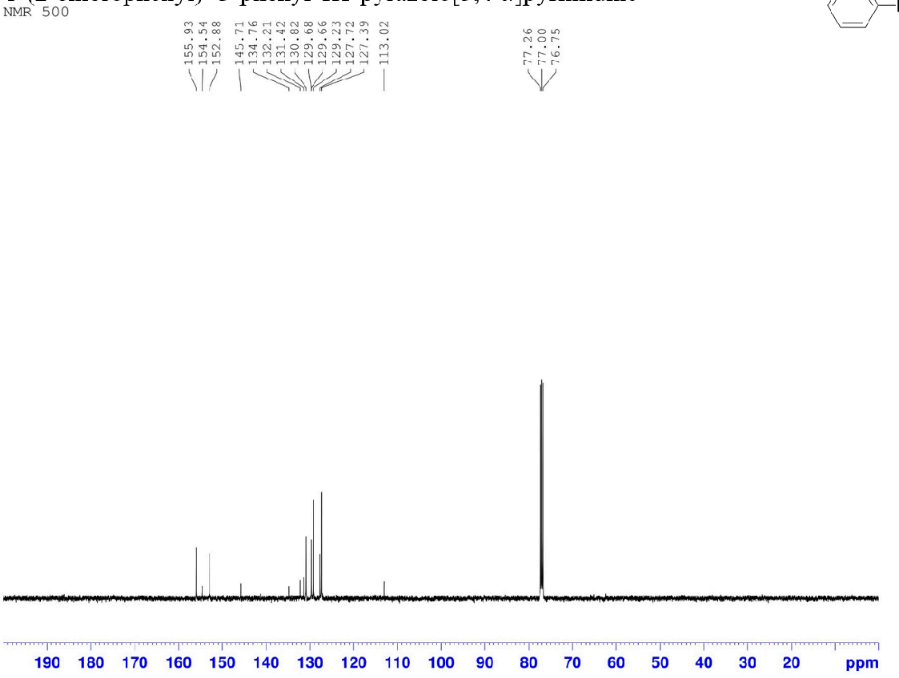

3d

1-(3-Methylphenyl) -3-phenyl-1*H*-pyrazolo[3,4-*d*]pyrimidine

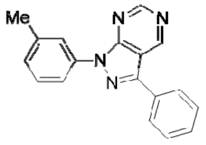

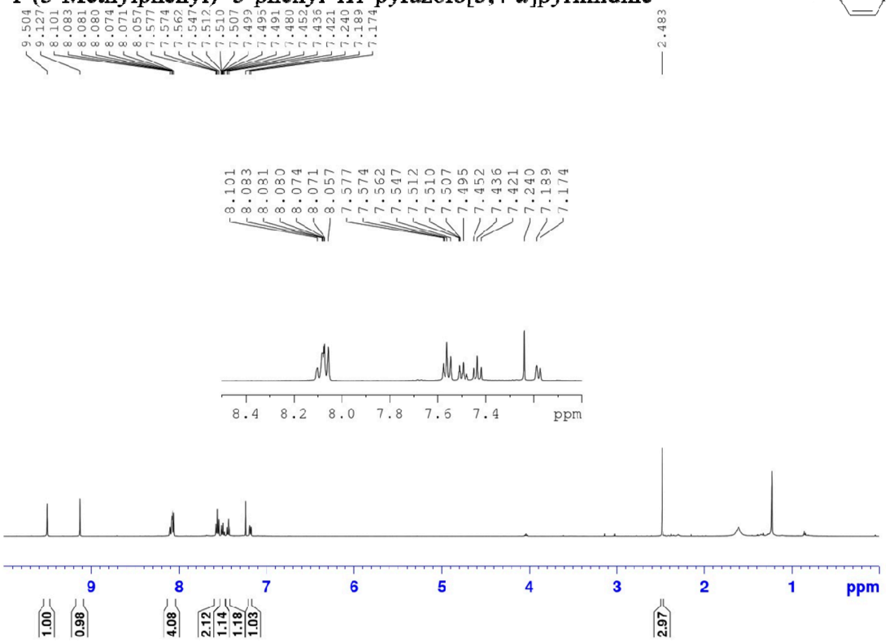

3d

1-(3-Methylphenyl) -3-phenyl-1*H*-pyrazolo[3,4-*d*]pyrimidine

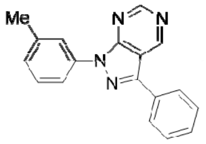

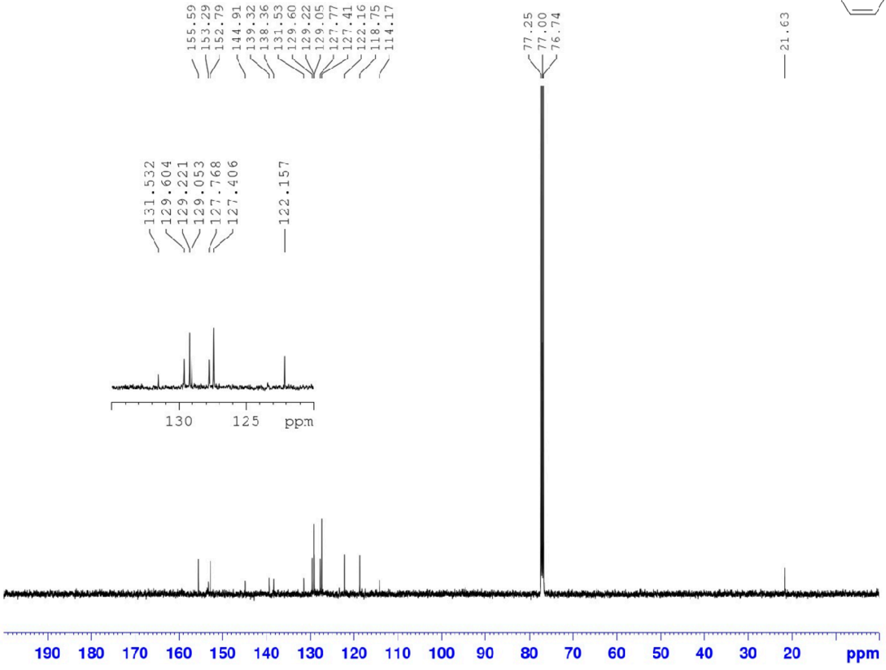

3e

1-(3-chlorophenyl) -3-phenyl-1*H*-pyrazolo[3,4-*d*]pyrimidine

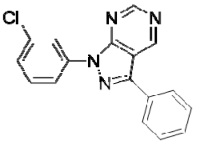

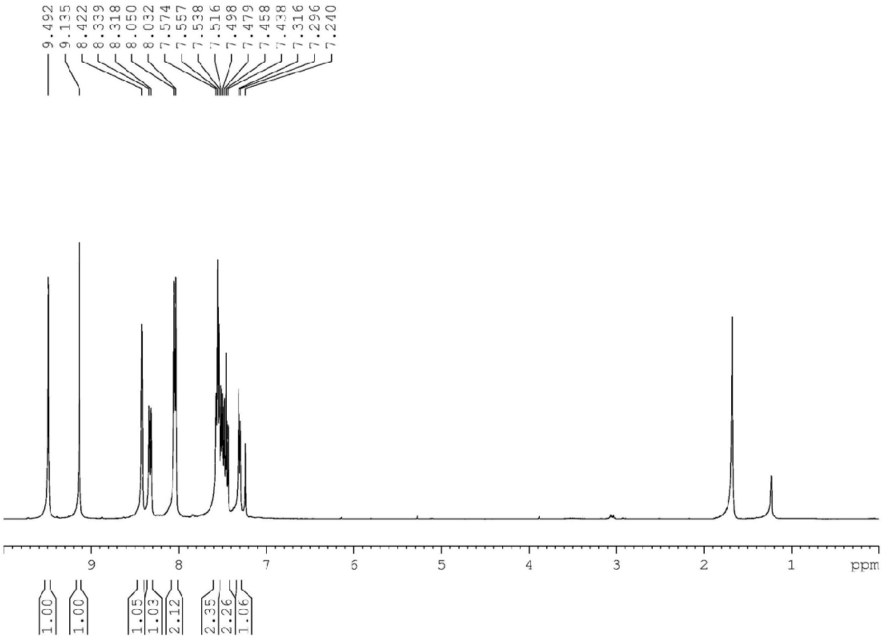

3e

1-(3-chlorophenyl) -3-phenyl-1*H*-pyrazolo[3,4-*d*]pyrimidine

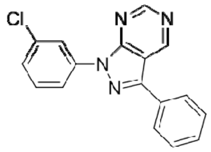

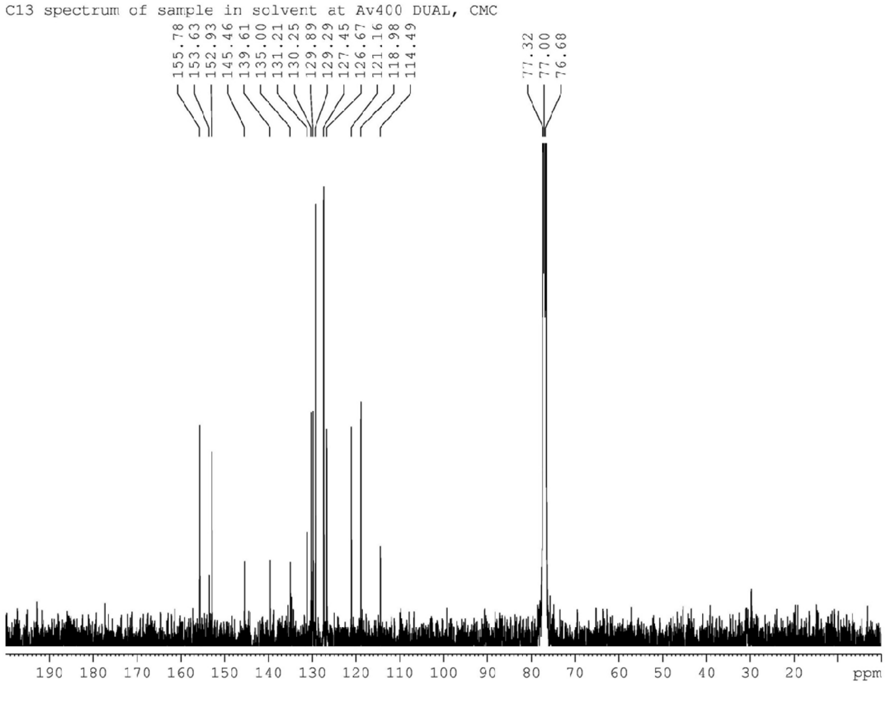

3f

1-(3-Nitrophenyl) -3-phenyl-1*H*-pyrazolo[3,4-*d*]pyrimidine

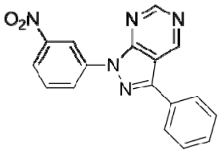

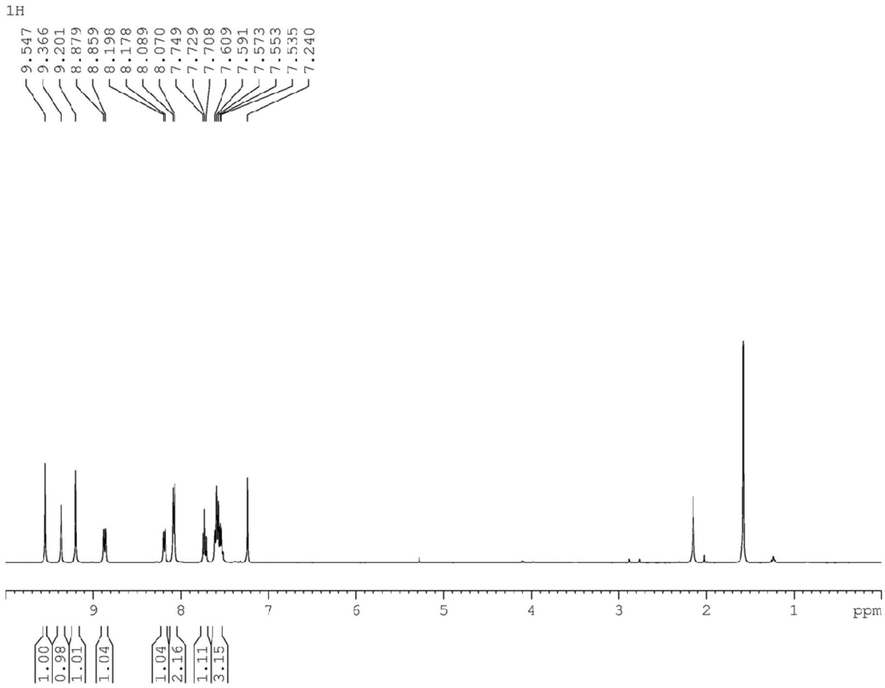

3f

1-(3-Nitrophenyl) -3-phenyl-1*H*-pyrazolo[3,4-*d*]pyrimidine

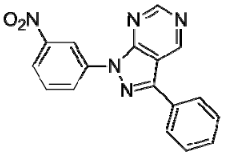

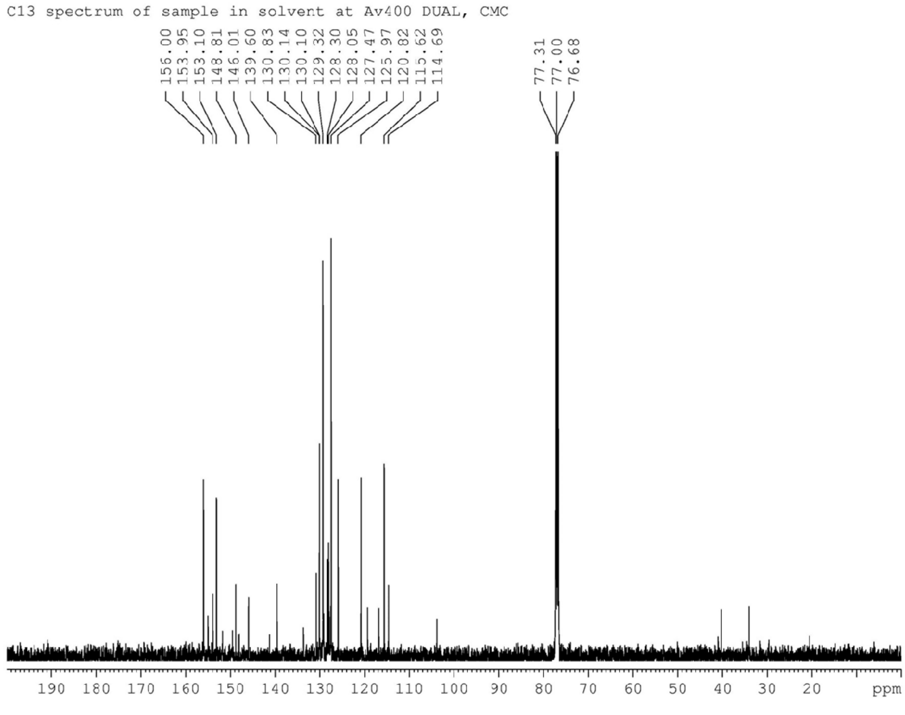

3g

1-(4-Methylphenyl) -3-phenyl-1*H*-pyrazolo[3,4-*d*]pyrimidine

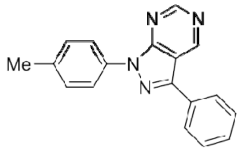

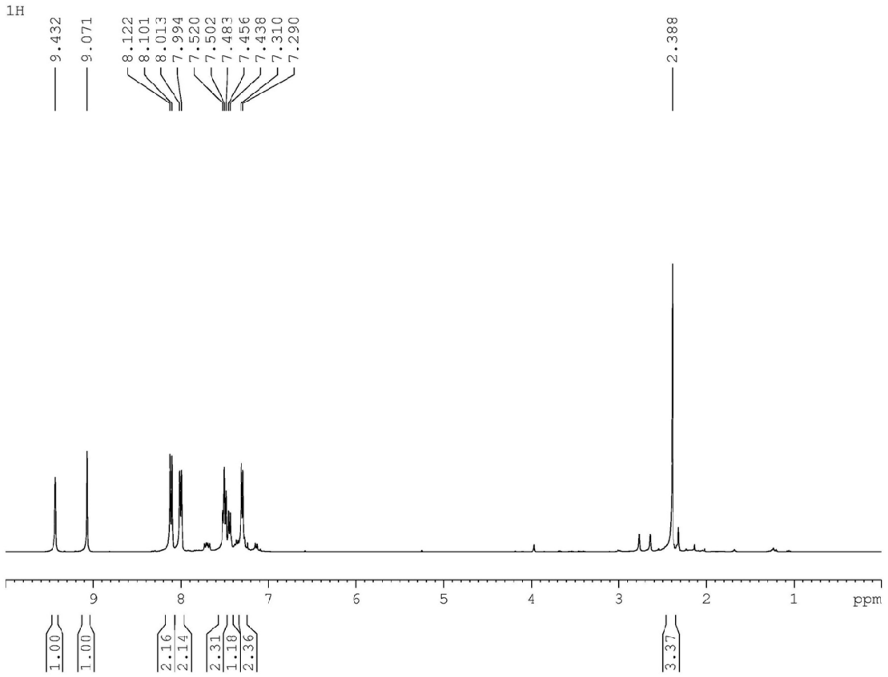

3g

1-(4-Methylphenyl) -3-phenyl-1*H*-pyrazolo[3,4-*d*]pyrimidine

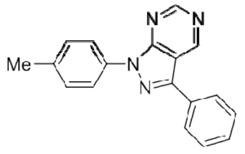

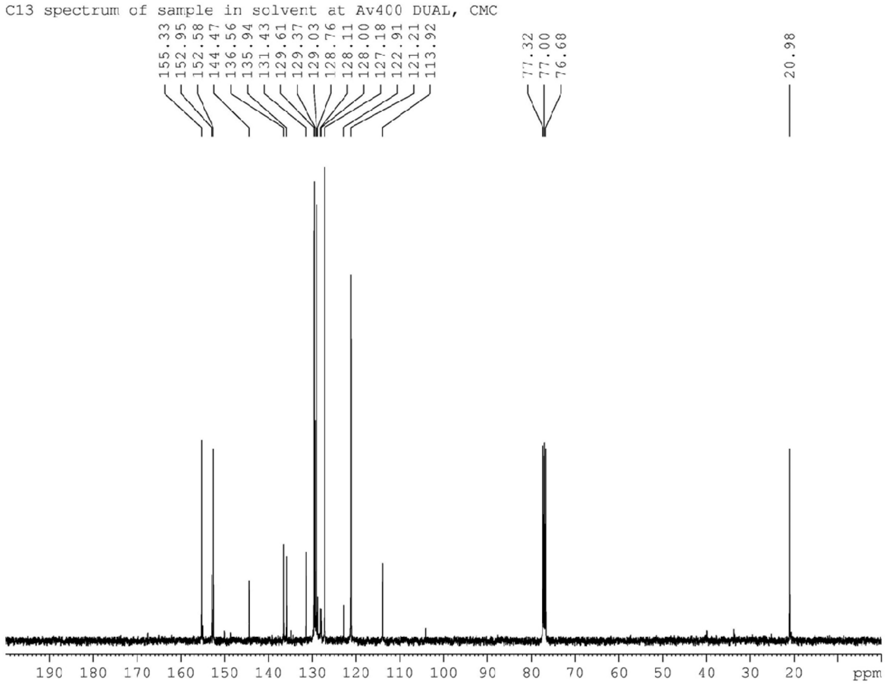

3h

1-(4-chlorophenyl) -3-phenyl-1*H*-pyrazolo[3,4-*d*]pyrimidine

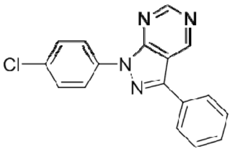

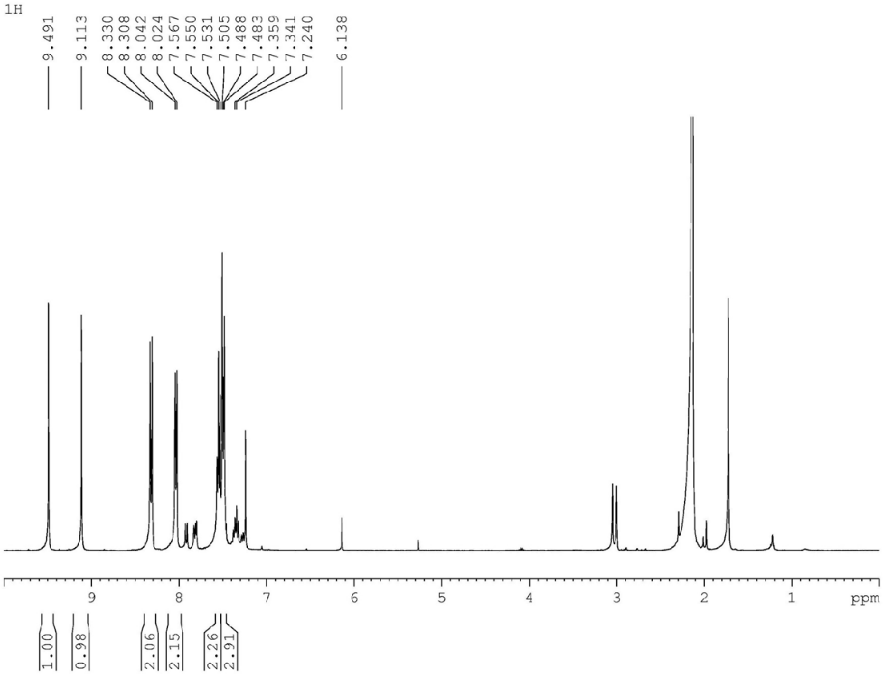

3h

1-(4-chlorophenyl) -3-phenyl-1*H*-pyrazolo[3,4-*d*]pyrimidine

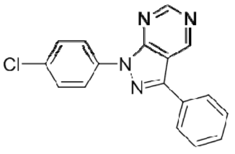

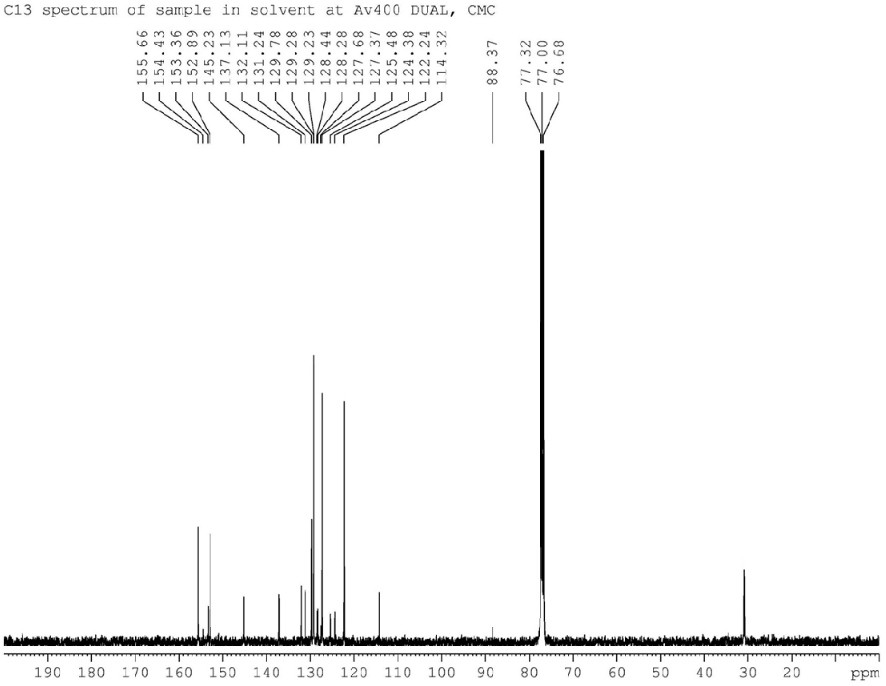

3i

1-(4-Bromophenyl) -3-phenyl-1*H*-pyrazolo[3,4-*d*]pyrimidine

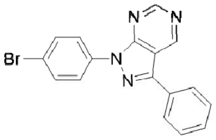

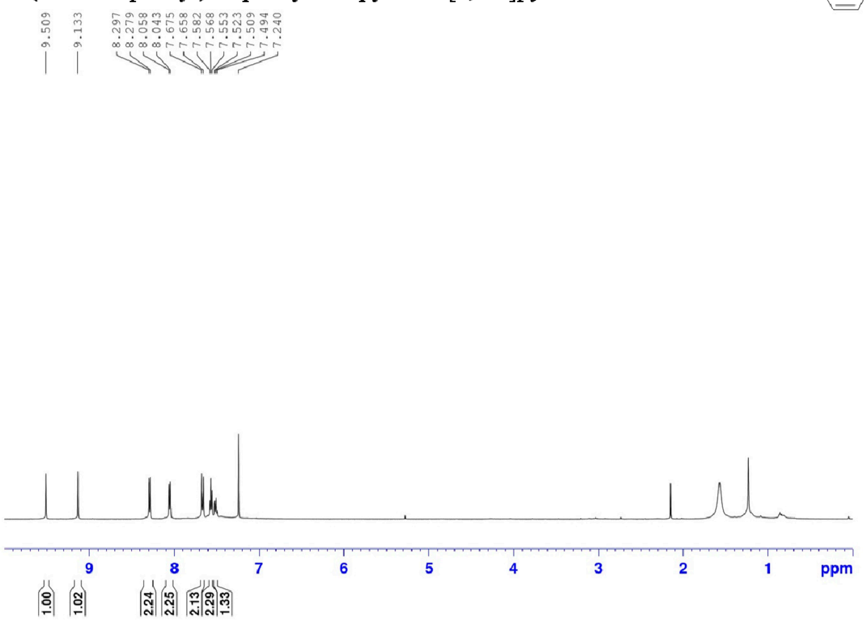

3i

1-(4-Bromophenyl) -3-phenyl-1*H*-pyrazolo[3,4-*d*]pyrimidine

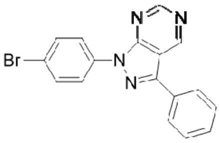

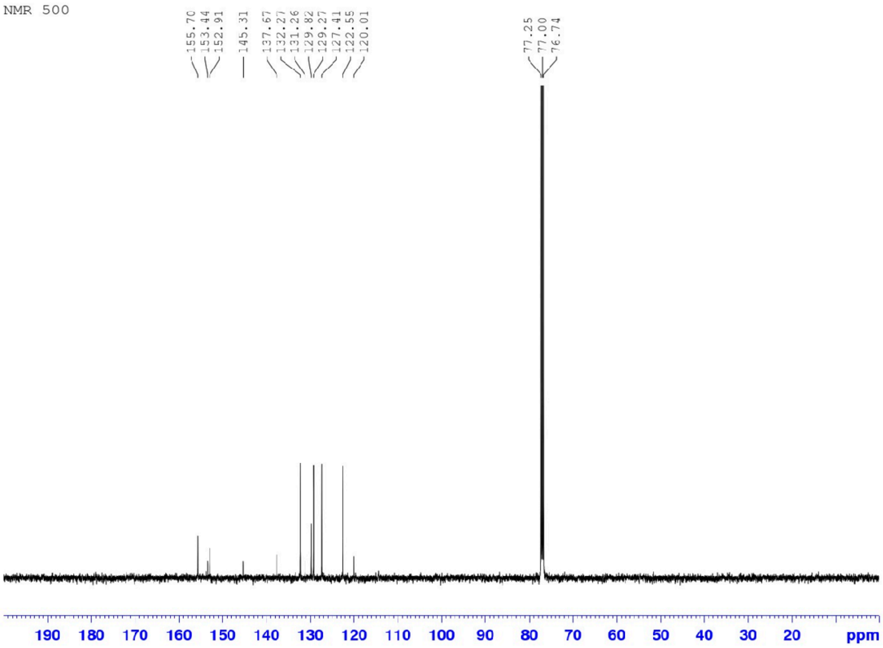

3j

3-Methyl-1-phenyl-1*H*-pyrazolo[3,4-*d*]pyrimidine

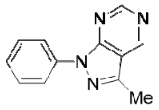

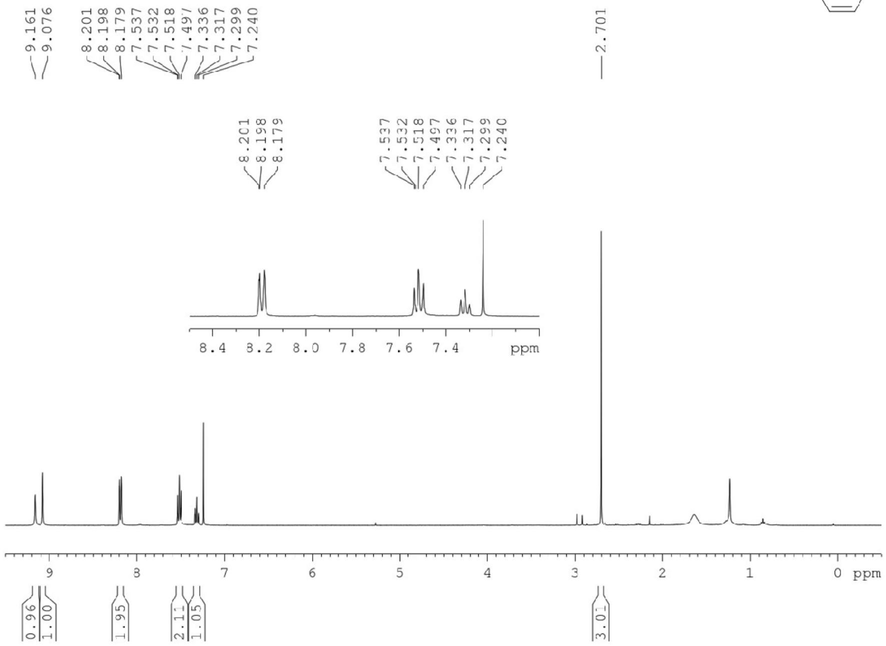

3j

3-Methyl-1-phenyl-1*H*-pyrazolo[3,4-*d*]pyrimidine

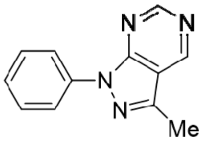

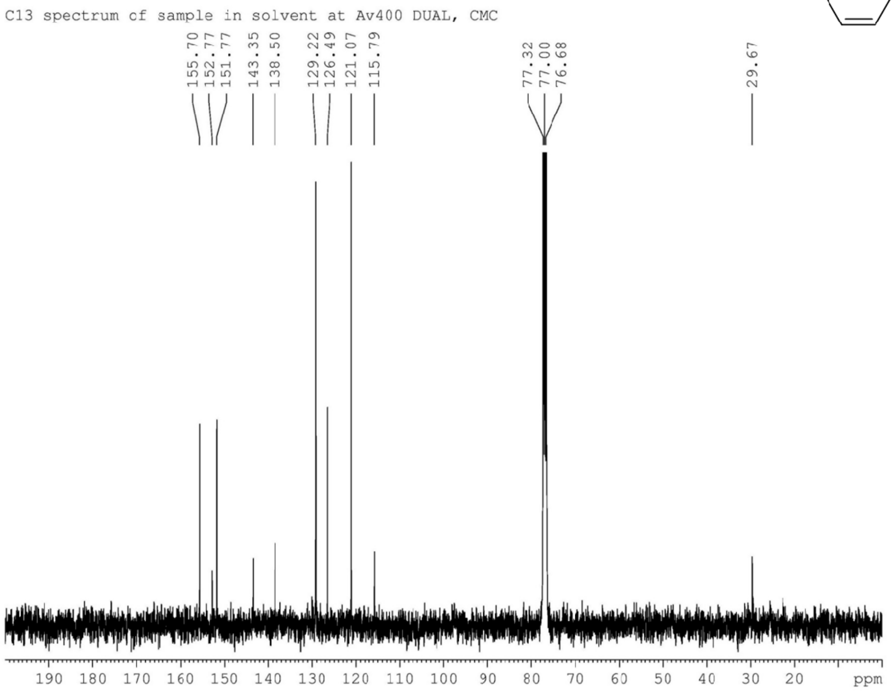

3k

3-tert-butyl-1-phenyl-1*H*-pyrazolo[3,4-*d*]pyrimidine

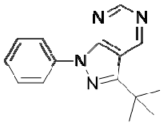

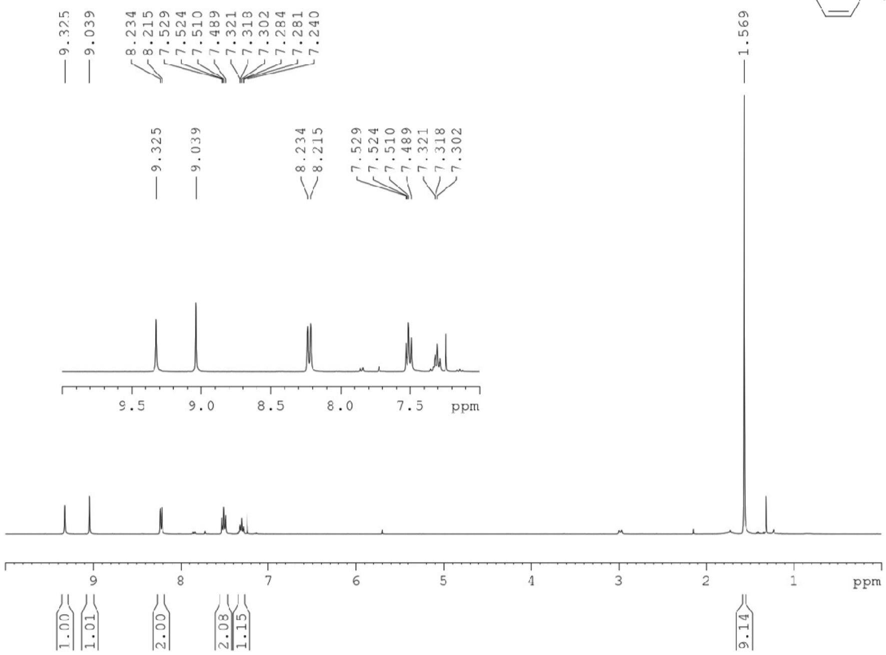

3k

3-tert-butyl-1-phenyl-1*H*-pyrazolo[3,4-*d*]pyrimidine

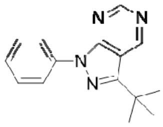

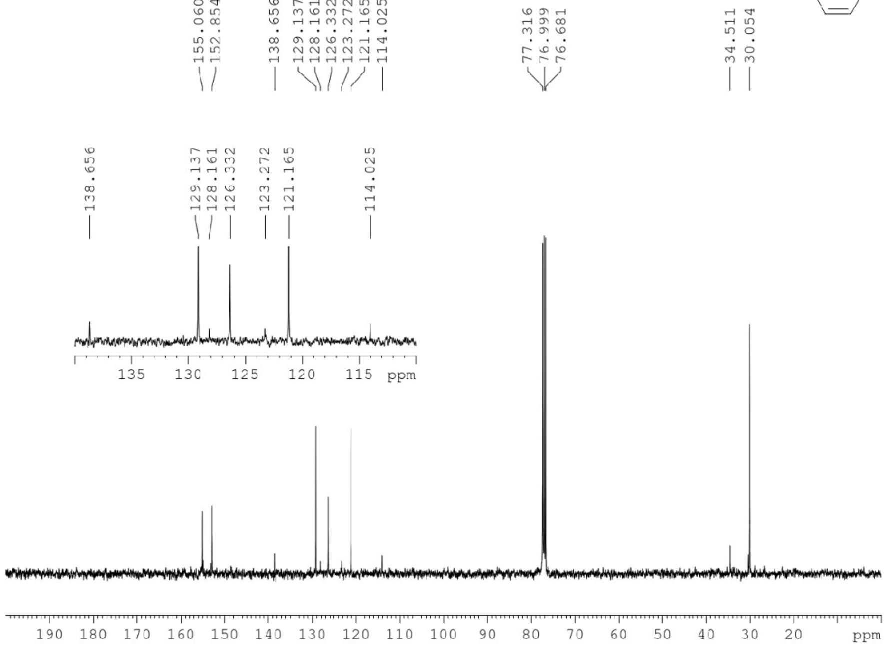

3l

3-(4-Mcthylphcnyl)-1-phcnyl-1*H*-pyrazolo[3,4-*d*]pyrimidinc

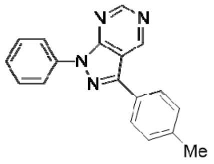

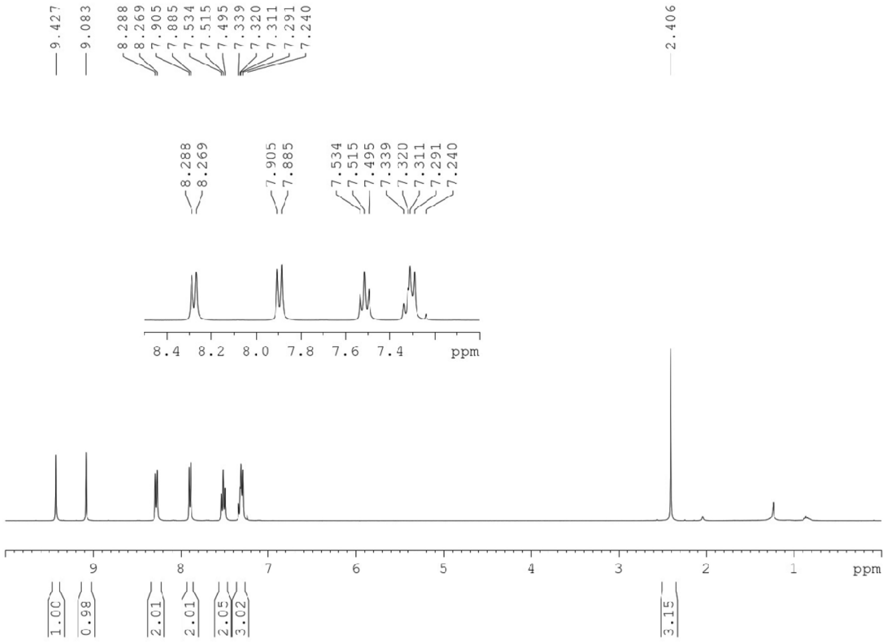

3l

3-(4-Mcthylphcnyl)-1-phcnyl-1*H*-pyrazolo[3,4-*d*]pyrimidinc

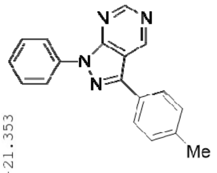

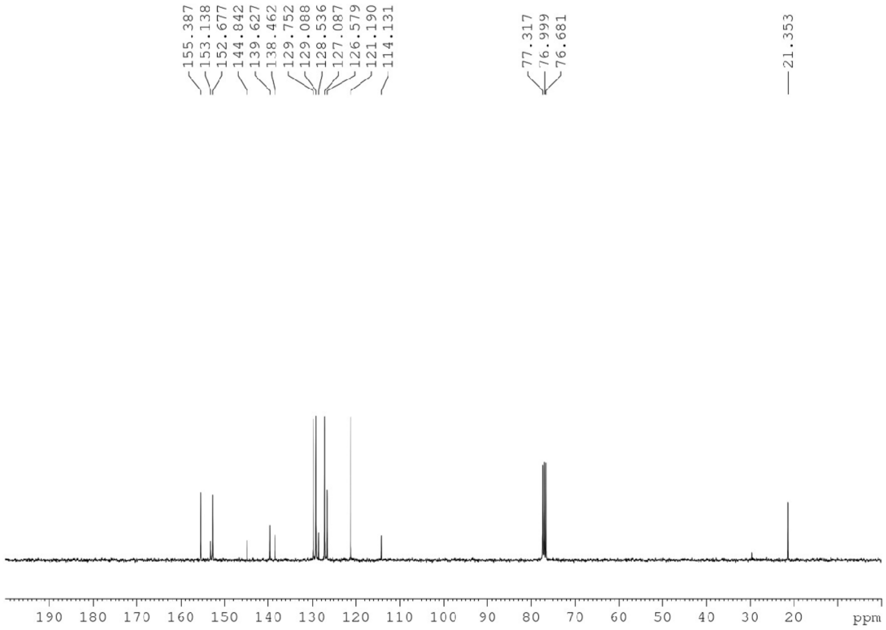

3m

3-(4-Chlorophenyl)-1-phenyl-1*H*-pyrazolo[3,4-*d*]pyrimidine

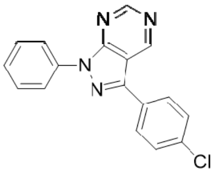

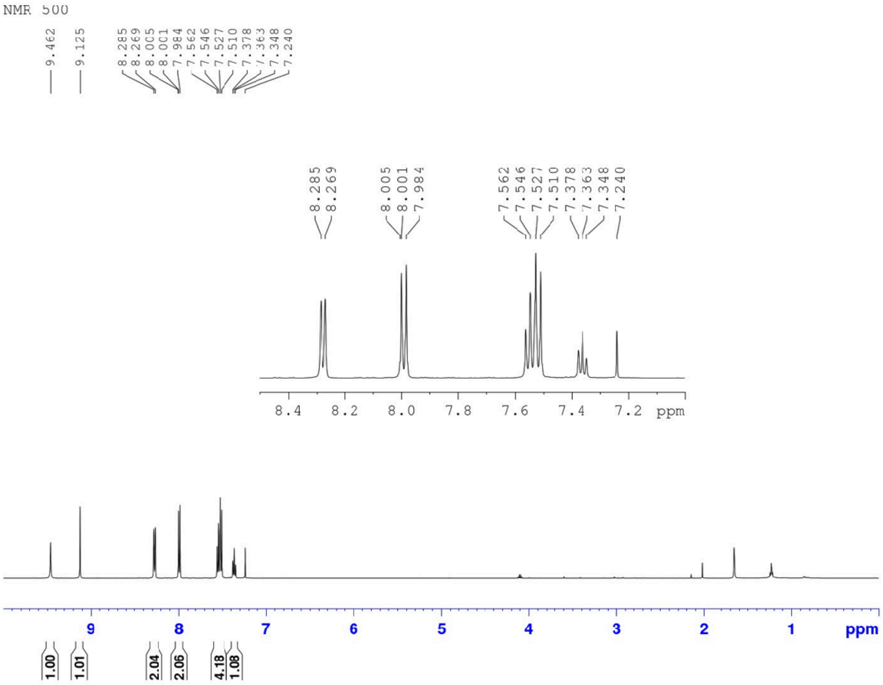

3m

3-(4-Chlorophenyl)-1-phenyl-1*H*-pyrazolo[3,4-*d*]pyrimidine

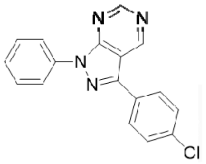

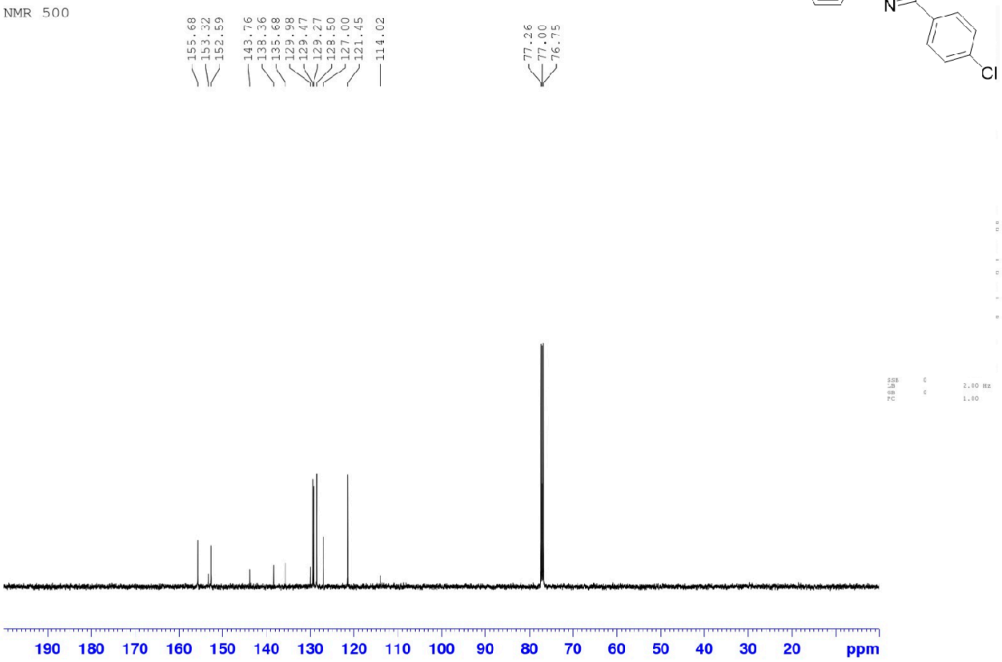

3n

3-(4-Methoxylphenyl)-1-phenyl-1*H*-pyrazolo[3,4-*d*]pyrimidine

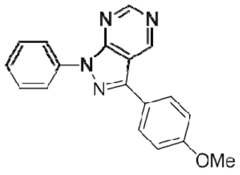

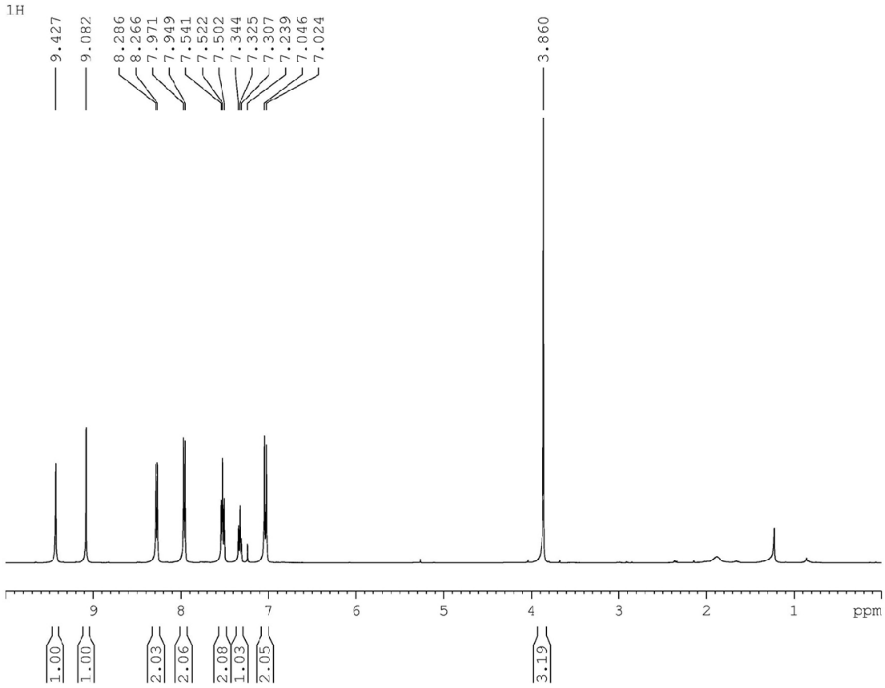

3n

3-(4-Methoxylphenyl)-1-phenyl-1*H*-pyrazolo[3,4-*d*]pyrimidine

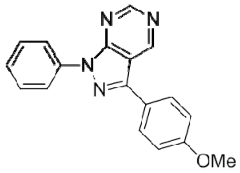

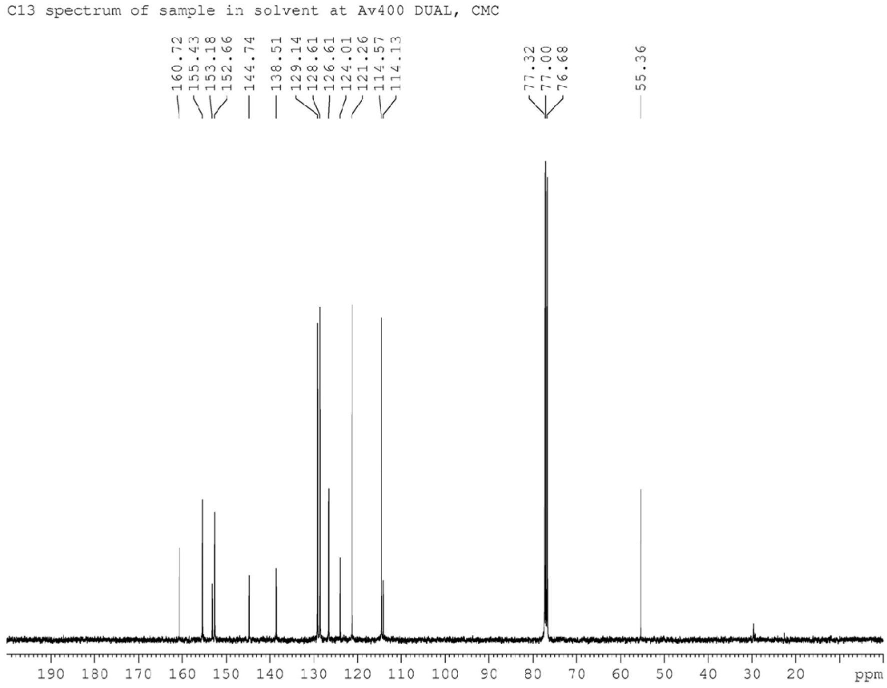

Supplement: Supplementary file 1 [file molecules-22-00820-s001.pdf]
